# Tai Chi exercise reduces circulating levels of inflammatory oxylipins in postmenopausal women with knee osteoarthritis: results from a pilot study

**DOI:** 10.3389/fmed.2023.1210170

**Published:** 2023-08-16

**Authors:** Chwan-Li Shen, John W. Newman, Moamen M. Elmassry, Kamil Borkowski, Ming-Chien Chyu, Chanaka Kahathuduwa, Volker Neugebauer, Bruce A. Watkins

**Affiliations:** ^1^Department of Pathology, Texas Tech University Health Sciences Center, Lubbock, TX, United States; ^2^Center of Excellence for Integrative Health, Texas Tech University Health Sciences Center, Lubbock, TX, United States; ^3^Center of Excellence for Translational Neuroscience and Therapeutics, Texas Tech University Health Sciences Center, Lubbock, TX, United States; ^4^United States Department of Agriculture, Agricultural Research Service, Western Human Nutrition Research Center, Davis, CA, United States; ^5^Department of Nutrition, University of California, Davis, Davis, CA, United States; ^6^West Coast Metabolomics Center, Genome Center, University of California, Davis, Davis, CA, United States; ^7^Department of Molecular Biology, Princeton University, Princeton, NJ, United States; ^8^Department of Medical Engineering, Texas Tech University, Lubbock, TX, United States; ^9^Department of Neurology, Texas Tech University Health Sciences Center, Lubbock, TX, United States; ^10^Department of Psychiatry, Texas Tech University Health Sciences Center, Lubbock, TX, United States; ^11^Department of Pharmacology and Neuroscience, Texas Tech University Health Sciences Center, Lubbock, TX, United States; ^12^Garrison Institute on Aging, Texas Tech University Health Sciences Center, Lubbock, TX, United States

**Keywords:** Tai Chi, oxylipins, postmenopausal women, knee osteoarthritis, endocannabinoids, brain

## Abstract

**Background:**

Tai Chi (TC) controls pain through mind–body exercise and appears to alter inflammatory mediators. TC actions on lipid biomarkers associated with inflammation and brain neural networks in women with knee osteoarthritic pain were investigated.

**Methods:**

A single-center, pre- and post-TC group (baseline and 8 wk) exercise pilot study in postmenopausal women with knee osteoarthritic pain was performed. 12 eligible women participated in TC group exercise. The primary outcome was liquid chromatography tandem mass spectrometry determination of circulating endocannabinoids (eCB) and oxylipins (OxL). Secondary outcomes were correlations between eCB and OxL levels and clinical pain/limitation assessments, and brain resting-state function magnetic resonance imaging (rs-fMRI).

**Results:**

Differences in circulating quantitative levels (nM) of pro-inflammatory OxL after TC were found in women. TC exercise resulted in lower OxL PGE_1_ and PGE_2_ and higher 12-HETE, LTB_4_, and 12-HEPE compared to baseline. Pain assessment and eCB and OxL levels suggest crucial relationships between TC exercise, inflammatory markers, and pain. Higher plasma levels of eCB AEA, and 1, 2-AG were found in subjects with increased pain. Several eCB and OxL levels were positively correlated with left and right brain amygdala-medial prefrontal cortex functional connectivity.

**Conclusion:**

TC exercise lowers pro-inflammatory OxL in women with knee osteoarthritic pain. Correlations between subject pain, functional limitations, and brain connectivity with levels of OxL and eCB showed significance. Findings indicate potential mechanisms for OxL and eCB and their biosynthetic endogenous PUFA precursors that alter brain connectivity, neuroinflammation, and pain.

**Clinical Trial Registration:**

ClinicalTrials.gov, identifier: NCT04046003.

## Introduction

Knee osteoarthritis (OA), a progressive disease characterized by joint degeneration and inflammation (1), is manifested by movement limitations and chronic pain. Alleviating the pain and improving dysfunction in patients with knee OA are public health priorities. Symptomatic treatment is the aim of pharmacological management for knee OA pain relief.

While the etiology of knee OA pain and underlying causes are complex, compelling evidence suggests that oxylipins (OxL) and endocannabinoids (eCB) are involved in the progression of knee OA pain. The endocannabinoid system (ECS) has emerged as a possible therapeutic target for OA pain reduction (2, 3). The ECS is composed of eCB, at least 2 cannabinoid receptors [namely, cannabinoid receptor type 1 (CB1R) and cannabinoid receptor type 2 (CB2R)], their endogenous ligands [anandamide (AEA), 2-arachidonoylglycerol (2-AG)], other structurally related compounds, and the enzymes responsible for eCB biosynthesis and inactivation. eCBs are lipid mediators biosynthesized from polyunsaturated fatty acids (PUFA) and produced in the brain and peripheral tissues that mimic the action of Δ9-tetrahydrocannabinol (4). The ECS participates in pathophysiological processes such as pain, emotion, and memory function (3, 5, 6). In addition, the ECS can mediate pain and modulate knee OA symptoms (3, 5). The ECS may exert analgesic effects on OA pain as the eCB are found in cells of the nervous system responsible for pain processing as well as in immune cells that regulate the neuro-immune interactions to convey inflammatory hyperalgesia (7). CB1R and CB2R are found in the central nervous system (CNS) (8) including multiple sites in the brain (9); they are targets for neuroinflammation and neuropathic pain (7).

Many of the eCB and OxL are biosynthesized from PUFA of the essential omega-6 and omega-3 families (10), although endocannabinoid-like compounds are derived from saturated fatty acids. The dietary levels of the different families of omega-6 and omega-3 PUFA directly influence the extent of which eCB and OxL are synthesized, thereby influencing the levels of pro-inflammatory or less inflammatory OxL (11).

Expression of CB2R protein by both neurons and microglia in the spinal cord was significantly increased in an OA pain model, suggesting that targeting eCB may have potential in treating OA (12). In human studies, individuals with OA pain compared with healthy individuals without OA pain had increased levels of 2-AG in plasma and CB1R and CB2R gene expression in peripheral blood lymphocytes, and these changes were correlated with pain, emotion, and cognitive symptoms (5).

OxLs are recognized lipid-derived mediators of inflammation and immune cell function in patients with OA pain (13). For example, relative to those without OA pain, individuals with OA pain had higher levels of circulating cyclooxygenase-2 (COX-2) products, 8-iso-PGF2α (14), 15-keto-dihydroPGF2α (14), PGE2 (15), and 15-LOX product 15-HETE (15), suggesting higher systemic inflammation and OxL production.

The eCB system (ECS) is a strategic factor for synaptic plasticity and homeostatic processes of the nervous system (9) including the receptor ligands (AEA and 2-AG), that participate in synaptic plasticity (16) and neuroinflammation. The eCBs affect mood, alter neurotrophin levels, restore mPFC output cognitive function, and inhibit pain (10, 17, 18). The actions of eCB and OxL which modulate pain and inflammation are driven at least in part by their actions on the amygdala (18). In contrast, moderate-intensity exercise increases eCB in the brain and blood and are thought to contribute to athletic wellbeing (10, 11) and play a role as retrograde synaptic messengers (16) for pain reduction.

Tai Chi (TC) exercise, a form of mind–body moderate-intensity exercise, affords both physical benefits for knee OA (physical function, balance, and muscle strength) (19) and a cognitive component promoting psychological wellbeing, life satisfaction, and improved perceptions of health (20, 21). A recent meta-analysis evaluated the effects of TC on walking function and posture control in elderly with knee OA and reported that (i) the length of the TC intervention time ranged from 8 to 24 weeks and the types of TC included Yang-style and Sun-style; and (ii) TC is effective and safe for knee OA (19). The authors conclude that TC can be used as an adjuvant, reliable physical training strategy to improve walking function and balance control (19). Similar to the recent review (19), the present study employed 24-form Yang-style TC for 8 weeks in older women with knee OA and measured clinical outcomes of pain, stiffness, and functional limitation using Western Ontario and McMasters Universities Osteoarthritis Index (WOMAC), Osteoarthritis Index, Visual Analog Scale (VAS), and Brief Pain Inventory (BPI) (22). Different from the review of previous studies (19), our investigation determined the effects of TC on circulating levels of eCB and OxL and their correlations with our reported clinical outcomes and amygdala-medial prefrontal cortex (mPFC) connectivity by resting-state functional magnetic resonance imaging (rs-fMRI) and diffusion tensor imaging (DTI) (22).

Chronic pain is thought to alter brain reward circuitry (23, 24) to include effects on shifting emotional states associated with pain chronification (25). Understanding this relationship between TC and reducing pain measurements of eCB and OxL can help advance knowledge to treat knee OA. Therefore, the objectives of this new study were (i) to explore if 8 weeks of TC exercise would modify plasma eCB and OxL levels and (ii) to conduct secondary analysis on correlations between TC-associated pre-post changes between eCB and OxL levels, clinical outcomes (pain, stiffness, and functional limitation), and amygdala mPFC connectivity changes. We hypothesize that changes in eCB levels and pattern as well as reduced inflammatory OxL mediators are, in part, responsible for the beneficial effects of TC on knee OA pain reduction and related changes in brain connectivity.

## Methods

### Study design

The present study was based on a single group pre-test and post-test design in 12 subjects to examine the effects of an 8 week TC exercise intervention on circulating eCB and OxL levels in postmenopausal women with knee OA. Sex is a strong risk factor for knee OA. After the age of 50, there is steep increase in incidence of knee OA in women compared with men, leading to a higher prevalence in women (26). Also, women with knee OA are more often accompanied by knee OA-associated pain and disability compared with men (26). Therefore, postmenopausal women with knee OA were recruited for this study.

Blood samples were collected at baseline and after 8 weeks of TC intervention. After centrifugation, plasma samples were obtained and stored at −80°C for later eCB and OxL analyses. This study was approved by the Institutional Review Board at the Texas Tech University Health Sciences Center (ClinicalTrials.gov Identifier: NCT04046003). In the correlation analyses of bioactive lipids and clinical outcomes 9 subjects were used. The criteria of knee OA exhibited symptoms was based on American College of Rheumatology clinical classification criteria for OA. Correlations between eCB and OxL levels and clinical outcomes (pain, stiffness, and functional limitation) data and between eCB and OxL levels and rs-fMRI data were also evaluated. Subjects were requested to maintain their current diets, medication, and physical activity, and not take any new supplements or vitamins.

### Recruitment of participants

In brief, postmenopausal women (≥50 years old) with knee pain were recruited from clinics and community centers in the Lubbock, Texas (United States) area by flyers and advertisements through newspaper (22). Inclusion criteria included (1) Postmenopausal women, (2) WOMAC pain score with at least 2 items out of 5 items are rated as moderate, severe, or extreme, respectively, (3) English literacy, (4) able to undergo an MRI scan for subjects having MRIs, (5) current pain in the knee, and (6) medical diagnosis of knee OA or knee(s) exhibited symptoms based on American College of Rheumatology clinical classification criteria for OA [as previously described Shen et al. (22)]. Exclusive criteria included (1) Prior experience with mind–body practice (e.g., TC, Qi Gong, yoga, or acupuncture) or physical therapy programs for knee OA within the past 3 months, (2) Severe medical limitations (i.e., dementia, symptomatic heart or vascular disease, or recent stroke) precluding full participation, (3) Medical/neurological or other systemic diseases affecting the musculoskeletal systems (i.e., polio/Parkinson’s/multiple sclerosis, rheumatoid arthritis, uncontrolled gout, etc. in addition to cerebral vascular accident or stroke) and diabetes with peripheral neuropathy affecting their sensory/balance, (4) intra-articular steroid injection or reconstructive surgery on most severely affected knee in the past 3 months, (5) intra-articular hyaluronic acid injections on most severely affected knee in the past 6 months, and (6) inability to walk without an assistive device.

### Tai Chi intervention

In this study the participants were enrolled in an 8 week 24-form Yang style TC program (22). Instructor led TC group consisted of 3 classes per week on 3 nonconsecutive days for 60 min at the Gym for the Department of Kinesiology and Sport Management, Texas Tech University, Lubbock. Compliance of TC classes was assessed by attendance records maintained by a Master TC instructor for the entire period. The Master TC instructor and assistant closely observed all participants and insured that each aspect of TC was performed correctly. The subjects did not have any TC practice experience prior to the study.

### Outcomes measures

#### Clinical outcomes

Details of the patient recruitment and screening, TC intervention and compliance, self-reported clinical outcomes assessed by Western Ontario and McMaster Universities Osteoarthritis Index (WOMAC: 5 items for pain, 2 items for stiffness, and 17 items for functional limitation), Visual Analog Scale (VAS), Brief Pain Inventory (BPI), and rs-fMRI data have been published previously (22).

#### Endocannabinoids, oxylipins extraction and UPLC-MS/MS analysis

Twenty four plasma samples (50 μL) (baseline and after TC) were subjected to protein precipitation in the presence of isotopically labeled analytical surrogates, filtered, and stored at −20°C until analysis as previously reported (27). After sample re-randomization, analytical targets were separated by UPLC and detected by electrospray ionization with positive/negative switching and multiple reaction monitoring on an API 6500 QTrap (AB Sciex, Framingham, MA, United States). Analytes were quantified using surrogate/analyte response ratios and 6- to 10-point calibration curves (27). Data were processed with AB Sciex MultiQuant v 3.0.1. All auto-integrations were reviewed and adjusted as necessary by an experienced analyst blinded to the sample key and reviewed by the project quality assurance officer. Data below the lowest calibration point or with >25% blank contribution were flagged and manually inspected, but all values were reported if signal was observed in >15% of the analyzed samples. Samples were processed in a single experimental batch along with two method blanks, and one plasma standard reference materials (NIST-1950; Sigma-Aldrich, St. Louis, MO), and accuracy was evaluated by comparison to laboratory historical measures of these materials (26). All values are quantitative in nM. It should be noted that monoacylglycerol isomerization occurs under these conditions, and the sum of the 1- and 2-isomers should only be considered as a surrogate measure of changes in 2-AG tone.

### Data processing and statistical analyses

Descriptive statistics were calculated to inspect the distributional properties of eCB and OxL. Further, values for eCB and OxL are also presented as standardized differences calculated from the difference between values of treatment and control, divided by the pooled SEM (28, 29). All analyses were conducted using R statistical software version 4.0.5 (30). Wilcoxon signed-rank tests were performed to examine the changes between pre- and post-TC intervention for eCB and OxL metabolites. Statistical significance was determined at 0.05 alpha level and an effect size was computed for each comparison. Furthermore, Spearman’s correlation analyses were performed using these parameter estimates between pre- vs. post-intervention changes in eCB and OxL and the corresponding changes in clinical measures (i.e., WOMAC, VAS, BPI, and fMRI). A final analysis of Spearman’s correlation of data was performed for the eCB and OxL values for all clinical measures and fMRI. Partial least squares discriminate analysis (PLS-DA) was used to visualize metabolite differences in women using endocannabinoid and oxylipin data at baseline (week 0) and after (week 8) TC exercise. Analyses were performed using auto-scaled data after the imputation of metabolites missing was detected in >70% of study participants (31). All metabolites were used to perform the analysis. Metabolites with variable importance in projections (VIP) >1 were considered significant in group discrimination. VIP scores were correlated with the *t*-test value of *p*s.

## Results

### Participants

Table 1 shows the demographic information and health status of all participants as previously reported (22). The subjects were recruited locally around the city of Lubbock Texas in the United States. The women had no adverse effects from TC exercise.

**Table 1:** Demographic characteristics of study participants.

Variables	Subjects (*n* = 12)
Age [*y*]	64.5 ± 6.7
Weight [kg]	82.5 ± 10.2
Height [cm]	164.3 ± 6.3
Body mass index [kg/m^2^]	30.6 ± 4.1
Regular physical activities [*n* (%)]	9 (75)
Low (walking, gardening)	4 (44.4)
Medium (bicycling, swimming)	5 (55.6)
High (aerobic, running)	0 (0)
Medical history questions [*n* (%)]	
General health rated “good”	12 (100)
Total knee replacement	2 (16.7)
History of rheumatoid arthritis or gout	1 (8.3)
Heart value/cardiac bypass surgery, stent procedure, or pacemaker	2 (16.7)
History stroke or heart attack	1 (8.3)
Low back pain and/or leg pain	2 (16.7)
Hormone or hormone-like therapy use	2 (16.7)
Blood pressure drug use	3 (25)
Thyroid hormone drug use	2 (16.7)
Pain medication use	4 (33.4)
Calcium/vitamin D use	6 (50)
History of cigarettes (current or ever-smoked)	3 (25.0)
Alcohol consumption	7 (58.3)

All data are mean ± standard deviation unless otherwise specified and adapted from reference [Shen et al. (22)].

### Endocannabinoids and oxylipins

Values for plasma levels of eCB AEA, 1,2-AG, and DHEA were lower based on standardized differences after TC exercise compared to baseline (Table 2). Relative to baseline, the subjects had lower plasma levels for LEA after 8 week TC. Notably, AEA was not higher after TC a condition of moderate exercise in contrast to other studies in subjects (10). It is important to note that a dietary record was not obtained from the women in this investigation, yet the baseline and after TC measurements of relevant eCB and OxL precursor substrate PUFAs did not differ (value area ratio means ± SD, baseline and post-TC, arachidonic acid 5.64 and 5.47 ± 2.20, eicosapentaenoic acid 5.20 and 5.92 ± 3.76, docosahexaenoic acid 5.36 and 5.75 ± 3.96 area ratios). Several eCB and OxL levels were highly positively correlated with both left and right brain amygdala-mPFC functional connectivity in subjects.

**Table 2:** Effect of TC on endocannabinoids (eCB) and related-compounds levels in plasma of women with OA.

eCB	Substrate fatty acid	Baseline	8 weeks TC	*p*-value	Standardized difference
sum of 1,2 AG	Arachidonic acid	245 ± 65	172 ± 25	0.652	−1.05
sum of 1,2 LG	Linoleic acid	5,660 ± 829	4,860 ± 910	0.57	−0.653
sum of 1,2 OG	Oleic acid	4,340 ± 1,380	3,800 ± 702	0.652	−0.351
AEA	Arachidonic acid	2.68 ± 0.29	2.10 ± 0.33	0.113	−1.32
αLEA	Linoleic acid	0.18 ± 0.02	0.20 ± 0.04	0.93	0.46
DHEA	Docosahexaenoic acid	1.35 ± 0.22	1.14 ± 0.22	0.258	−0.68
LEA	Linoleic acid	4.81 ± 0.37	3.75 ± 0.64	0.0503*	−1.41
OEA	Oleic acid	10.6 ± 1.4	8.47 ± 0.71	0.387	−1.34
PEA	Palmitic acid	6.82 ± 2.39	6.49 ± 1.80	0.945	−0.11
SEA	Stearic acid	3.86 ± 0.41	3.87 ± 0.43	1.0	0.02

Units are nM. Values are means ± SEM of *n* = 12, baseline and 8 week Tai-Chi intervention in subjects with knee osteoarthritis. *Indicates *p* < 0.05. Other values are presented as standardized differences calculated from the difference between values of TC treatment and control, divided by the pooled SEM. AEA, N-arachidonoylethanolamine; 1-AG, 1-arachidonoylglycerol; 2-AG, 2-arachidonoylglycerol; DHEA, docosahexaenoyl ethanolamide; LEA, N-linoleylethanolamine; αLEA, alpha N-linoleylethanolamine; OEA, oleoylethanolamide; 2-LG, 2- linoleoylglycerol; 1-OG, 1-oleoylglycerol; 2-OG, 2-oleoylglycerol; PEA, palmitoylethanolamide; SEA, N-stearoylethanolamine.

TC resulted in significant changes for many OxL compared to pre-TC (baseline) in women (Table 3). OxL derived from arachidonic acid, prostanoids PGE_1_ and PGE_2_, were reduced after TC. The reduced level of pro-inflammatory PGE_2_ is a clear indication that TC reduced this potent pro-inflammatory OxL. The levels of 12-HEPE and 12,13-DiHODE (metabolites of eicosapentaenoic and linolenic acids, respectively) as well as 12-HETE, 20-HETE, and LTB_4_ (metabolites of arachidonic acid) in subjects were higher post TC. The increase in LTB_4_ could be due to higher inflammation or macrophage activation with TC (32). The not significant values of other identified OxL are presented in Supplementary Table S1.

**Table 3:** Significant effects of TC on plasma oxylipins levels in women with osteoarthritis.

OxL	Substrate fatty acid	Baseline	8 weeks TC	*p*-value	Standardized difference
PGE1	Dihomo-γ-linolenic acid	0.59 ± 0.14	0.16 ± 0.06	0.0159*	−2.96
PGE2	Arachidonic acid	15.08 ± 5.69	2.13 ± 0.65	0.036*	−2.41
12,13-DiHODE	α-Linolenic acid	0.81 ± 0.36	2.37 ± 1.07	0.0625#	1.38
12-HEPE	Eicosapentaenoic acid	0.32 ± 0.06	1.95 ± 0.93	0.00078*	1.75
12-HETE	Arachidonic acid	6.24 ± 1.08	18.50 ± 5.46	0.0625#	2.20
20-HETE	Arachidonic acid	5.47 ± 1.04	7.38 ± 1.54	0.0907#	1.07
LTB4	Arachidonic acid	0.04 ± 0.02	0.37 ± 0.14	0.0424*	1.71
Protectin DX	Eicosapentaenoic acid	0.16 ± 0.06	0.40 ± 0.08	0.077#	2.27

Units are nM. Values are means ± SEM of *n* = 12, baseline and 8 week Tai-Chi intervention in subjects with knee osteoarthritis. *Indicates *p* < 0.05. #Indicates 0.05 < *p* < 0.1. Other values are presented as standardized differences calculated from the difference between values of TC treatment and control, divided by the pooled SEM. DiHODE, dihydroxy octadecadienoic acid; HEPE, hydroxy eicosapentaenoic acid; HETE, hydroxy eicosatetraenoic acid; LT, leukotriene; PG, prostaglandin.

In general, many products of arachidonic acid can support pro-inflammatory actions compared to those derived from the substrate eicosapentaenoic acid. Interestingly TC resulted in lower LEA compared to baseline. The higher blood levels of 12-HETE and 12-HEPE likely indicate greater 12-LOX pathway flux supported by inflammation (33). The full names and metabolic pathways for the synthesis of OxL are described elsewhere (31, 34).

**Figure 1:**
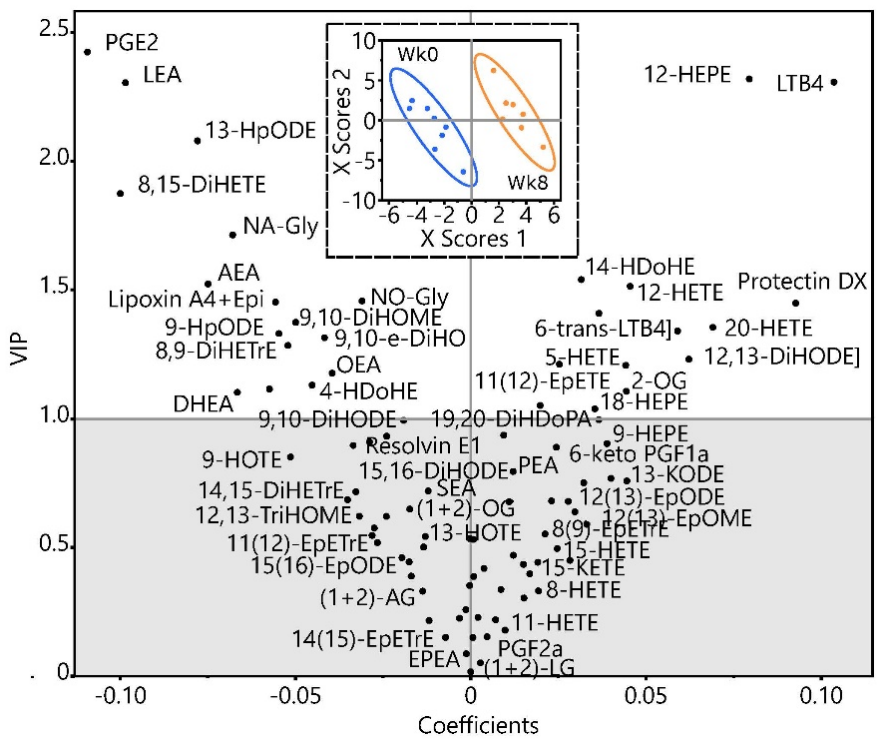
Partial least squares discriminate analysis of endocannabinoid and oxylipin data in women at baseline week 0 (Wk 0) and after 8 weeks (Wk 8) of TC exercise.

Figure 1 is the partial least squares discriminate analysis (PLS-DA) for all eCB and OxL data from subjects at baseline week 0 (Wk 0) and after week 8 (Wk 8) of TC exercise. All values are numerically presented in Supplementary Table S2. The PLS-DA shows that values in the upper left quadrant are of high variable importance, VIP above 1 (*p* ≤ 0.05) and negative relationship with TC exercise. The positive values associated with TC exercise appear in the right upper quadrant of Figure 1. Thus, the VIP values show that TC reduced OxL PGE_2_, 8,9-DiHeTrE, and 8,15-DiHETE and eCB AEA, which are all derivatives of arachidonic acid, but led to higher 12-HEPE (EPA derivative), and LTB_4_ and 12-HETE (both arachidonate derivatives). Moreover, for the measured variables many of the eCB and OxL showed strong VIP values, negative or positive relationships. Several other eCB and OxL measurements revealed weaker VIP values. The weaker VIP values suggest a lower confidence in their apparent actions in TC exercise, but do not mean a lesser role of TC on subjects and relationships to pain and well-being. It is interesting that eCB DHEA derived from DHA showed a VIP score of greater than one and might suggest that DHA could help attenuate inflammation or serve in another capacity during TC. Based on VIP values, numerous OxL-derived from long-chain n-6 and n-3 PUFA were changed and thus may play a role in mediating the effects of TC exercise.

Analyses in Figure 1 were performed on data (in nM) after imputation of missing values by visit for metabolites with >60% of measured metabolites, followed by transformation to normal distributions. The analysis scores plot (inset) shows group discrimination with data for each group, baseline, and post-TC, bound by bivariate normal ellipses (*p* = 0.95). The variable coefficient plot shows metabolite strength in discrimination. Metabolites with variable importance in projections (VIP) >1 are considered to have discriminating power. Due to the low subject number, leave one out cross validation was used to build the model. Group discrimination was achieved with a minimum of 2 dimensions as shown (*Q*^2^ = 0.45; *r*^2^*x* = 0.24, *r*^2^*y* = 0.96) but was improved considering a third dimension (*Q*^2^ = 0.64; *r*^2^*x* = 0.36; *r*^2^*y* = 0.99). A complete list of metabolites, their concentration group means, VIP scores, and value of *p*s from 2-tailed *t*-tests are included in Supplementary Table S2.

### Correlations between pain, functional limitation, stiffness, brain connectivity, and plasma levels of OxL and eCB

Another analysis revealed significant correlations (Spearman’s correlation coefficient) between pain assessment and circulating levels of OxL and eCB, suggesting important relationships between inflammatory/anti-inflammatory markers and pain assessment as shown in Table 4. The correlation comparisons are all measurements for pre- and post-TC. All pain assessments [BPI, brief pain inventory; VAS, visual analog scale; Western Ontario and McMaster Universities Osteoarthritis Index (WOMAC)] correlations for OxL and eCB and eCB-like compounds were significant based on Spearmen’s correlation coefficient. In general, most OxL values from pre- and post-TC exercise showed a negative correlation to pain-related assessments. Therefore, differences for pre- and post-TC values indicate a decline in most OxL that potentially plays a role in inflammation. However, positive correlations were found for 6-keto PGE_2_,15-keto PGE_2_, LTB_4_ (BPI_3: pain level at its worst in the past 24 h) with the pain assessments. In contrast, higher plasma eCB levels, such as DHEA (product of docosahexaenoic acid, omega-3 PUFA) were associated with less pain (BPI_9C: in the past 24 h, pain has interfered your walking ability) as were higher plasma levels of eCB-like compound LEA (BPI_5: pain level on average, BPI_9G: in the past 24 h, pain has interfered your enjoyment of life, BPI_9H: in the past 24 h, pain has interfered your ability to concentrate; VAS_3: what is the overall amount of pain you have experienced in the last week in your knee).

**Table 4:** Correlations between plasma OxL and eCB with clinical outcomes and functional aspects of brain connectivity.

Compound	Pain-associated parameters	Spearman’s correlation coefficient	*p*-value
PGE2	WOMAC Pain	−0.6778302	0.044809
15-Keto PGE2	WOMAC Pain	0.8170879	0.00717
13-KODE	WOMAC Pain	0.6861985	0.041242
9,10-e-DiHO	WOMAC Stiffness	−0.779773	0.013205
15,16-DiHODE	WOMAC Stiffness	−0.8475794	0.003909
9,10-DiHODE	WOMAC Stiffness	−0.8814826	0.001677
12-HETE	WOMAC Stiffness	−0.6865393	0.041101
12-HEPE	WOMAC Stiffness	−0.6865393	0.041101
13-HpODE screen	WOMAC Stiffness	−0.7882488	0.011614
15(16)-EpODE	WOMAC Stiffness	−0.7204425	0.028567
14(15)-EpETrE	WOMAC Stiffness	−0.7712972	0.014937
LEA	WOMAC Stiffness	−0.7543456	0.018842
αLEA	WOMAC Stiffness	−0.8136762	0.007622
15-Keto PGE2	WOMAC Functional Limitation	0.8984341	0.000994
PGF2α	WOMAC Functional Limitation	0.6833333	0.042442
9,12,13-TriHOME	WOMAC Functional Limitation	0.7333333	0.024554
8,9-DiHETrE	WOMAC Functional Limitation	0.75	0.019942
5,6-DiHETrE	WOMAC Functional Limitation	0.7166667	0.029818
9-HOTE	WOMAC Functional Limitation	0.75	0.019942
15-HEPE	WOMAC Functional Limitation	−0.7	0.03577
6-keto PGF1α	BPI_3	0.7532538	0.019115
LTB4	BPI_3	0.6667641	0.049822
9-KODE	BPI_4	−0.8231358	0.006414
17(18)-EpETE	BPI_4	−0.7798129	0.013197
6-trans-LTB4	BPI_5	−0.6738606	0.046568
19,20-DiHDoPA	BPI_5	−0.8157259	0.007348
13-HOTE	BPI_5	−0.7004603	0.035596
15-HETE	BPI_5	−0.9043918	0.000809
12-HETE	BPI_5	−0.8600589	0.002936
11-HETE	BPI_5	−0.8245925	0.006241
15-HEPE	BPI_5	−0.7625264	0.016882
12-HEPE	BPI_5	−0.8600589	0.002936
LEA	BPI_5	−0.7447932	0.021315
αLEA	BPI_5	−0.9398581	0.000166
9-HOTE	BPI_6	0.7235681	0.027557
12(13)-EpODE	BPI_6	0.6810052	0.043434
17(18)-EpETE	BPI_6	−0.6810052	0.043434
15-HETE	BPI_9A	−0.6668859	0.049765
11-HETE	BPI_9A	−0.7951332	0.01042
5-KETE	BPI_9A	−0.709635	0.032243
9(10)-EpOME	BPI_9A	−0.7780336	0.013549
17(18)-EpETE	BPI_9A	−0.6668859	0.049765
6-trans-LTB4	BPI_9B	−0.7576183	0.018041
9-HpODE screen	BPI_9B	0.7491057	0.020174
9(10)-EpOME	BPI_9B	−0.7916686	0.01101
14,15-DiHETrE	BPI_9C	−0.8398413	0.004611
5-HETE	BPI_9C	−0.6755245	0.045826
17-HDoHE	BPI_9C	−0.7302967	0.025464
9-KODE	BPI_9C	−0.7302967	0.025464
DHEA	BPI_9C	−0.8033264	0.009111
13-HOTE	BPI_9D	−0.7065429	0.033349
15-HETE	BPI_9D	−0.7065429	0.033349
11-HETE	BPI_9D	−0.7491057	0.020174
9(10)-EpOME	BPI_9D	−0.7746434	0.014236
15(16)-EpODE	BPI_9D	−0.7065429	0.033349
17(18)-EpETE	BPI_9D	−0.7661309	0.016063
αLEA	BPI_9D	−0.7235681	0.027557
6-trans-LTB4	BPI_9E	−0.7257286	0.026873
12,13-DiHODE	BPI_9E	−0.7082411	0.032739
9(10)-EpOME	BPI_9E	−0.8131657	0.007691
15(16)-EpODE	BPI_9E	−0.6994974	0.03596
αLEA	BPI_9E	−0.7519597	0.019441
PGF2α	BPI_9F	0.803683	0.009056
9-HOTE	BPI_9F	0.7010852	0.03536
17,18-DiHETE	BPI_9G	−0.68201	0.043004
15-HETE	BPI_9G	−0.6732663	0.046835
12-HETE	BPI_9G	−0.6732663	0.046835
12-HEPE	BPI_9G	−0.6732663	0.046835
9-HEPE	BPI_9G	−0.7082411	0.032739
14-HDoHE	BPI_9G	−0.9006029	0.000924
11(12)-EpETE	BPI_9G	0.6994974	0.03596
LEA	BPI_9G	−0.6732663	0.046835
17,18-DiHETE	BPI_9H	−0.7352845	0.023981
19,20-DiHDoPA	BPI_9H	−0.6668859	0.049765
13-HOTE	BPI_9H	−0.7267347	0.026558
15-HETE	BPI_9H	−0.8464321	0.004008
12-HETE	BPI_9H	−0.8207827	0.006701
11-HETE	BPI_9H	−0.7609339	0.017252
12-HEPE	BPI_9H	−0.8207827	0.006701
9-HEPE	BPI_9H	−0.709635	0.032243
14-HDoHE	BPI_9H	−0.7267347	0.026558
9(10)-EpOME	BPI_9H	−0.6754357	0.045865
17(18)-EpETE	BPI_9H	−0.7010852	0.03536
LEA	BPI_9H	−0.7951332	0.01042
αLEA	BPI_9H	−0.854982	0.003309
12,13-DiHOME	BPI_9I	−0.707475	0.033013
9,10-DiHOME	BPI_9I	−0.7302967	0.025464
9-HETE	BPI_9I	−0.7104413	0.031959
9-KODE	BPI_9I	−0.7302967	0.025464
13-HpODE screen	BPI_9I	−0.7302967	0.025464
9-KODE	VAS_1	−0.829925	0.005632
9-HpODE screen	VAS_1	0.8668106	0.002486
9(10)-EpODE	VAS_1	−0.6731614	0.046883
12,13-DiHODE	VAS_2	−0.7010852	0.03536
14-HDoHE	VAS_2	−0.6668859	0.049765
9-KODE	VAS_2	−0.6668859	0.049765
17(18)-EpETE	VAS_2	−0.6754357	0.045865
9,10-DiHOME	VAS_3	−0.7491057	0.020174
15,16-DiHODE	VAS_3	−0.6724927	0.047184
12,13-DiHODE	VAS_3	−0.7916686	0.01101
9,10-DiHODE	VAS_3	−0.7235681	0.027557
17,18-DiHETE	VAS_3	−0.7576183	0.018041
15-HETE	VAS_3	−0.6895178	0.03988
12-HETE	VAS_3	−0.7405932	0.022467
12-HEPE	VAS_3	−0.7405932	0.022467
LEA	VAS_3	−0.8257188	0.006109
αLEA	VAS_3	−0.8172063	0.007155
8,9-DiHETrE	VAS_4	0.6810052	0.043434
5,6-DiHETrE	VAS_4	0.6895178	0.03988
9-HOTE	VAS_4	0.8257188	0.006109

Compound	Brain connectivity measures	Spearman’s correlation coefficient	*p*-value
6-keto PGF1a	LA_mPFC	1	0
14,15-DiHETrE	LA_mPFC	0.9746794	0.004818
8,9-DiHETrE	LA_mPFC	0.9746794	0.004818
5,6-DiHETrE	LA_mPFC	0.9746794	0.004818
19,20-DiHDoPA	LA_mPFC	0.9746794	0.004818
13-HOTE	LA_mPFC	0.9746794	0.004818
11-HETE	LA_mPFC	0.9746794	0.004818
9-HETE	LA_mPFC	0.9210526	0.026311
5-KETE	LA_mPFC	0.9746794	0.004818
LEA	LA_mPFC	0.9746794	0.004818
DHEA	LA_mPFC	0.9746794	0.004818
8,9-DiHETrE	RA_mPFC	0.9	0.037386
5,6-DiHETrE	RA_mPFC	0.9	0.037386
13-HOTE	RA_mPFC	0.9	0.037386
12(13)-Ep-9-KODE	RA_mPFC	1	0
5-KETE	RA_mPFC	0.9	0.037386
LEA	RA_mPFC	0.9	0.037386
sum of LG	RA_mPFC	0.9	0.037386
Resolvin E1	LAD	0.9	0.037386
15,16-DiHODE	LAD	−0.9	0.037386
9-HpODE screen	LAD	−1	0
14(15)-EpETrE	LAD	−0.9	0.037386
11(12)-EpETrE	LAD	−0.9	0.037386
8(9)-EpETrE	LAD	−0.9	0.037386
sum of OG	LAD	−0.9	0.037386
15,16-DiHODE	RAD	1	0
9-HpODE screen	RAD	0.9	0.037386

Spearman correlation analysis were performed on values for eCB and OxL (*n* =9) in nM, and clinical pain, physical function and stiffness assessments (i.e., WOMAC, VAS, and BPI) (*n* =12), and brain functional magnetic resonance imaging (fMRI) measures of connectivity (*n* =7). All analyses are differences between pre and post Tai Chi to perform the correlations. BPI, brief pain inventory; VAS, Visual Analog Scale; Western Ontario and McMaster Universities Osteoarthritis Index (WOMAC). BPI_3, Pain level at its worst in the past 24 hours; BPI_4, Pain level at its least in the past 24 hours; BPI_5, Pain level on average; BPI_6, Pain level right now; BPI_9B, In the past 24 hours, pain has interfered your Mood; BPI_9C, In the past 24 hours, pain has interfered your Walking Ability; BPI_9D, In the past 24 hours, pain has interfered your normal work; BPI_9E, In the past 24 hours, pain has interfered your relations with other people; BPI_9F, In the past 24 hours, pain has interfered your sleep; BPI_9G, In the past 24 hours, pain has interfered your Enjoyment of life; BPI_9H, In the past 24 hours, pain has interfered your ability to concentrate; BPI_9I, In the past 24 hours, pain has interfered your appetite; VAS_1, What is the LEAST amount of pain you have experienced in the last week in your knee?; VAS_2, What is the MOST amount of pain you have experienced in the last week in your knee?; VAS_3, What is the OVERALL amount of pain you have experienced in the last week in your knee?; VAS_4, What is the amount of pain you are experiencing in your knee RIGHT NOW? LA_mPFC, L/amygdala-mPFC functional connectivity; LAD, L/amygdala-mPFC DTI connectivity; RA_mPFC, R/amygdala-mPFC functional connectivity; RAD, R/amygdala-mPFC DTI connectivity.

Several positive correlations were found between brain left and right amygdala-mPFC functional connectivity (LA_mPFC and RA_mPFC) and OxL and eCB (Table 4). For example, the 8,9-DiHETrE, 5,6-DiHETrE, and 5-KETE (arachidonic acid products) showed a positive correlation for both LA_mPFC and RA_mPFC functional connectivity. The eCB DHEA and LEA revealed positive correlations for LA_mPFC, and a positive relationship was found for LEA and sum of LG with RA_mPFC. Negative relationships were found for OxL and LAD. The higher blood levels of 12-HETE and 12-HEPE associated with WOMAC-stiffness and BPI-5: pain level on average, could suggest greater 12-LOX pathway flux supported by inflammation (33).

### Comparison of pre- and post-TC scatter plots for pain and brain connectivity measurements

**Figure 2:**
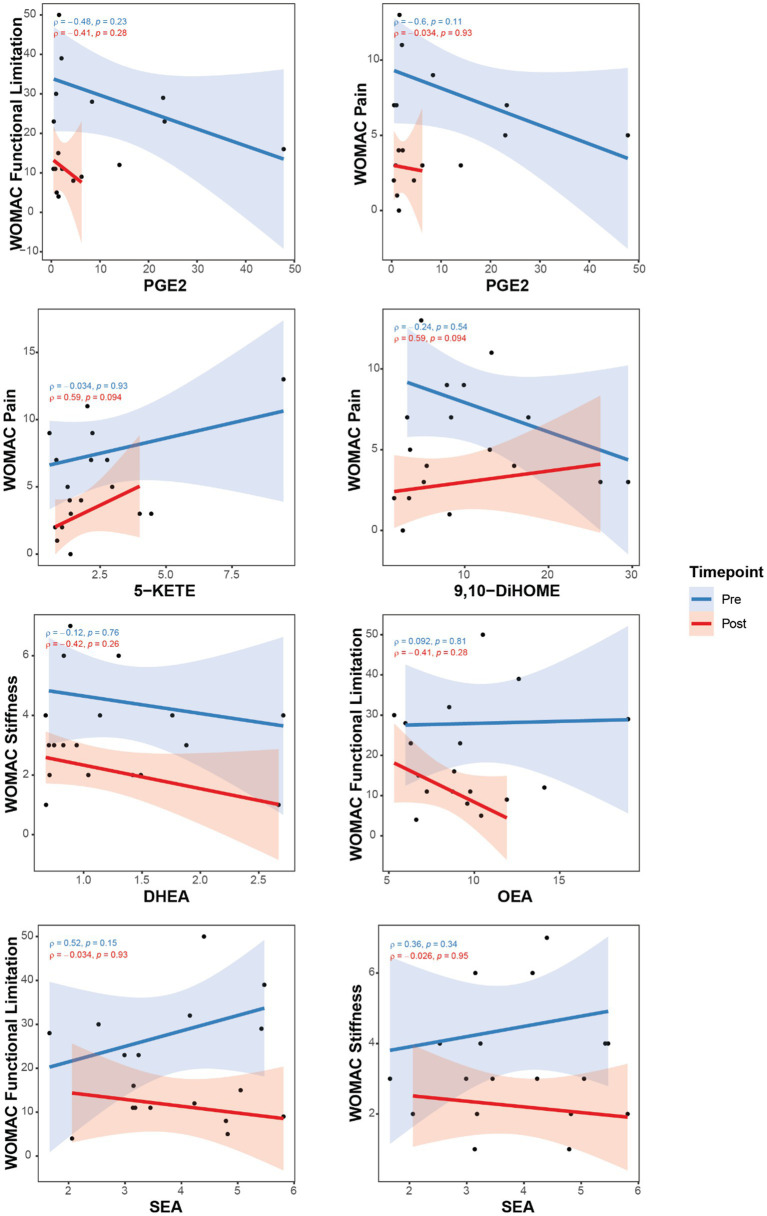
Correlations between pain assessments with OxL and eCB and related compounds for subjects pre- and post-TC exercise. Oxylipins, (PGE2, 5-KETE, and 9,10DiHOME); Endocannabinoid DHEA, docosahexaenoyl ethanolamide; Endocannabinoid-like OEA, oleoylethanolamide; PEA, palmitoylethanolamide; SEA, N-stearoylethanolamine. Western Ontario and McMaster Universities Osteoarthritis Index (WOMAC).

Additional correlations for changes in OxL and eCB were done as individual pre- and post-TC scatter plots to illustrate the effects of TC as these parameters relate to pain and brain connectivity. Figure 2 shows some examples, specifically differences after TC revealed negative correlations between pain and OxL. Interestingly, the plots show robust TC physiological adaptations for changing OxL levels (e.g., PGE_2_, 5-KETE) and decreasing subject variability for these compounds.

**Figure 3:**
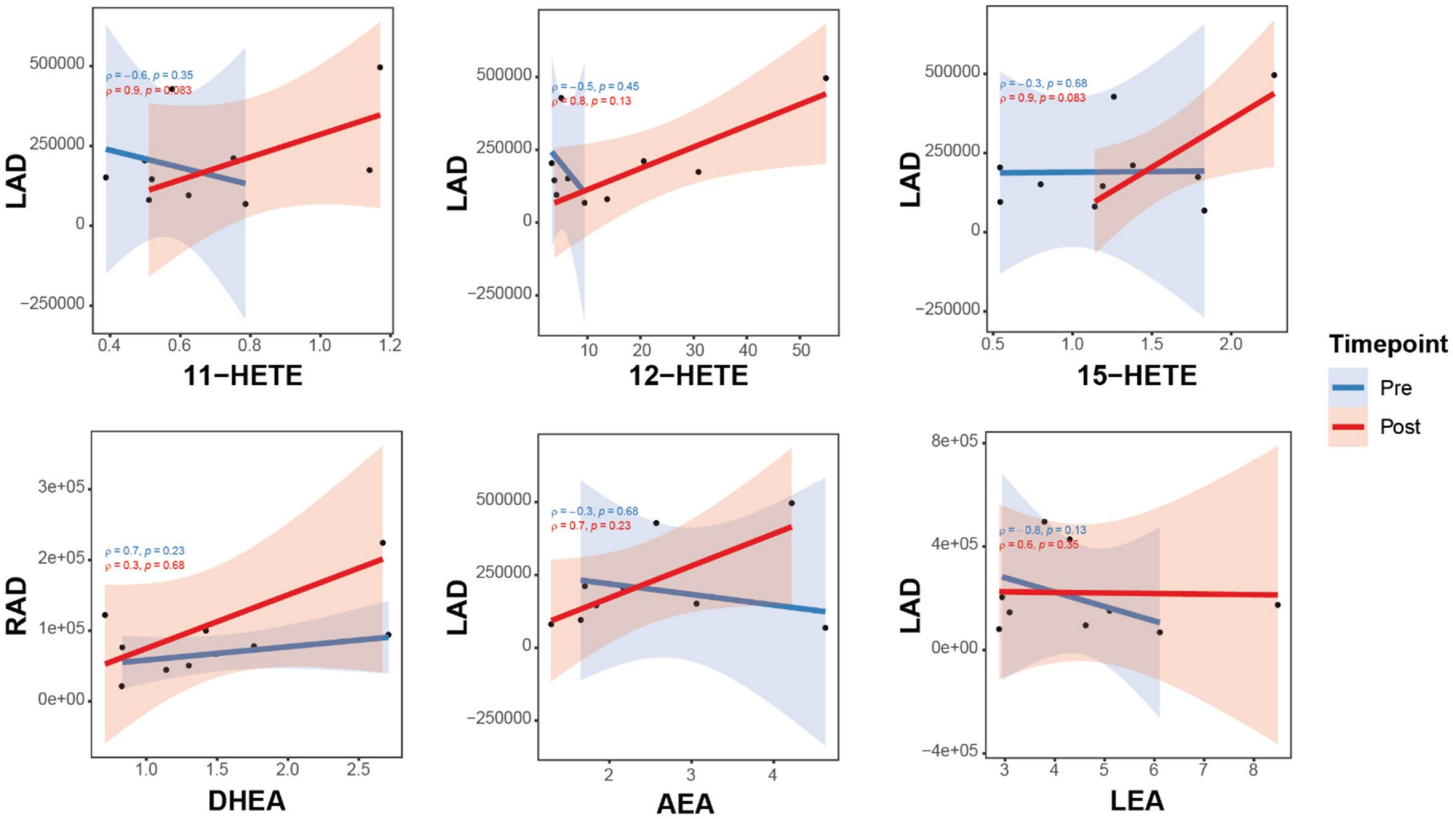
Correlations between brain connectivity with OxL and eCB and related compounds for subjects pre- and post-TC exercise. Brain connectivity measured as resting-state functional magnetic resonance imaging (rs-fMRI). Oxylipins (11-, 12-, 15-HETE, products of arachidonic acid). AEA, N-arachidonoylethanolamine; DHEA, docosahexaenoyl ethanolamide; LEA, N-linoleylethanolamine. LA_mPFC, L/amygdala-mPFC functional connectivity; LAD, L/amygdala-mPFC DTI connectivity; RA_mPFC, R/amygdala-mPFC functional connectivity; RAD, R/amygdala-mPFC DTI connectivity.

Consistent with significant correlations for differences in values presented in Table 4, the individual plots (Figures 2, 3) indicate a physiologic effect of TC to establish a lower threshold level of OxL in homeostasis. This new chemical threshold for OxL is likely responsible for lower WOMAC-pain values. With respect to eCB and eCB-like compounds, DHEA values were less variable in subjects after TC, which reflects a new level threshold change for the physiological adaptation from TC exercise (Figure 2). Also, OEA showed a tightening of values and negative slope for WOMAC-functional limitations and WOMAC-pain, as well as BPI-3: pain level at its worst in the past 24 h. SEA and WOMAC-functional limitation and stiffness showed an opposite slope post-TC compared to pre-TC or baseline (Figure 2).

The relationships of three HETE (11-, 12-, and 15-products of arachidonic acid) show a positive correlation with brain connectivity after TC (Figure 2). Moreover, the upward slope of all three HETE would suggest a strong association with changes in brain connectivity for LAD. This upward slope was also observed with the eCB DHEA and AEA for RAD and LAD, respectively (Figure 3). In contrast, the eCB-like compound LEA seems to change little with LAD values for brain connectivity after TC. However, LEA level was lower after TC (Table 2).

## Discussion

This pilot study is the first to examine the effects of 8 week TC intervention on circulating eCB and OxL levels in postmenopausal women with knee OA pain. The self-reported pain reduction and its impact on physical functioning results of WOMAC and BPI in OA subjects is evidence that 8 weeks of TC intervention diminishes pain, stiffness, and functional limitations that are linked to some extent with pro-inflammatory OxL biomarkers. The changes in lipid-derived eCB and OxL after TC are associated with improved well-being (11), which is important to pain perception, brain emotional circuits (25), and loss of gray matter density (35). Interestingly TC also resulted in lower LEA compared to baseline and may seem to lower activation of Transient Receptor Potential Vanilloid 1 (TRPV1) and pain sensation (36).

Oxidative stress and systemic low-grade inflammation are upregulated in OA (37). Thus, studies such as ours that focus on plasma eCB and OxL analyses are key to identifying biomarkers connected with OA progression. While previous studies (22, 38) examined the health benefits of TC intervention on mitigation of oxidative stress and inflammation in knee OA patients, no investigation has undertaken a systemic biological/physiological approach to determine the effects of TC on systemic levels of OxL and eCB. Herein, integration of the targeted metabolomics data and correlations with brain functional/structural connectivity and clinical data on pain, stiffness, and functional limitation assessment of our study affords new insights on TC as an intervention for pain management in OA patients. Further, the eCB and their lipid analogues are reported to influence oxidative stress (39). Our investigation is the first to examine the effect of TC exercise on an extensive plasma OxL and eCB lipid biomarker analyses, along with brain functional and structural connectivity in knee OA patients. Our results serve as a foundation for future mechanistic studies.

Our hypothesis examined an important knowledge gap on how TC improves pain associated with knee OA by measuring eCB and OxL as targets to alter inflammation in the peripheral and central nervous systems (11). We establish that TC changes circulating eCB and OxL levels, and amygdala mPFC connectivity, and reduces pain and stiffness, resulting in improved physical function. The effects of TC on OA patients have been evaluated using brain fMRI as described by Shen et al. (22) and Liu et al. (40, 41). Liu et al. reported that all exercises (Tai chi, Baduanjin, and stationary cycling) (i) significantly decreased right periaqueductal grey rs-functional connectivity with the medial orbital prefrontal cortex, and decreased rs-functional connectivity associated with improvements in knee pain; and (ii) significantly increased grey matter volume in the medial orbital prefrontal cortex. There was also significantly decreased rs-functional connectivity between the left ventral tegmental area and the medial orbital prefrontal cortex only in the Tai Chi and Baduanjin groups (40). From the same study, relative to the control group, all exercise groups (i) showed decreased rs-functional connectivity of the dorsolateral prefrontal cortex-supplementary motor area, (ii) increased rs-functional connectivity between the dorsolateral prefrontal cortex and anterior cingulate cortex, and (iii) increased grey matter volume in the supplementary motor area (41). Studies on TC and OA suggest TC can simultaneously modulate the resting-state functional connectivity of the descending pathway and reward/motivation system and blood inflammation markers (40, 41). TC is associated with a strengthening of functional and structural connectivity between the medial prefrontal cortex and left and right amygdala (22). Thus, TC effects are likely, in part, mediated by OxL and eCB as shown in the current study.

Another aspect of our clinical research is the relationship between dietary PUFA, which serves as substrate for the different groups of eCB and OxL, and as a consequence level of these inflammatory bioactive lipids to impact pain. We also found that the eCB DHEA which is derived from DHA showed a VIP score of greater than one, suggesting that dietary DHA could help attenuate inflammation or serve in another capacity during TC. Dietary fat is reported to influence plasma and intestinal concentrations of eCB, *N*-acylethanolamines (NAEs), and their precursors *N*-acylphosphatidylethanolamines (NAPEs) (42). Moreover, specific families of PUFA have long been recognized as substrate of pro-inflammatory and less inflammatory OxL (11). Thus, changing the amounts and types of n-3 and n-6 families of PUFA can lead to changes in less pro-inflammatory OxL, by replacing arachidonic acid with eicosapentaenoic acid to modulate inflammatory responses (10, 11, 43, 44). With respect to OxL in this study, the arachidonic acid product PGE_2_ was significantly lower and associated with lower pain post-TC, and the values for this OxL were less variable within subject group after TC. The higher levels of 12-HEPE (EPA derivative), and LTB_4_ and 12-HETE (both arachidonate derivatives) observed after TC are important since these OxL afford anti-inflammatory activity and modulate inflammatory response, respectively, via the PUFA substrate (33).

The effect of TC appears to stabilize OxL levels to a new physiological threshold for homeostasis. Considering these findings, future clinical research must include dietary PUFA and TC interactions on the amounts and types of circulating OxL and eCB (and eCB-like compounds) and on pain and brain connectivity. In support of our findings with TC, the actions of stretching were described to reduce inflammatory mediators and oxidative stress to lower pain (45). The authors emphasized the need to explain the anti-inflammatory properties of stretching for OA recovery, which is consistent with our hypothesis to understand the effects of TC and potential benefits causing changes in OxL and eCB levels post TC exercise to lower OA pain.

### Limitations and strengths

A limitation of this study is the small subject group; however, each subject was included for baseline and post TC treatment for analysis of eCB and OxL levels. The duration of TC was 8 weeks from baseline as two time points for the measurements. Nevertheless, this study confirms differences in pre- and post-TC exercise changes of the same OA subjects for a comprehensive panel of eCB and OxL levels in blood. These novel findings afford a new approach to understand exercise benefits on clinical biomarkers for OA. The application is two aspects of improving pain and wellbeing utilizing patient pain assessment and brain connectivity. TC altered levels of OxL derived from n-3 and n-6 PUFA family substrate and decreased pro-inflammatory states. The changes in brain connectivity post-TC and correlations with plasma eCB and OxL levels are noteworthy and suggest an important relationship with neuroinflammation and brain neuroplasticity for these lipid biomarkers under mind–body exercise. Future studies should examine the effects of dietary n-3 PUFA and include dietary records to ascertain if n-3 PUFA substrates alter the types of eCB, and further change pro-inflammatory OxL.

## Conclusion

Our findings identified an anti-neuroinflammatory role of TC exercise which is linked to modified blood levels of specific OxL, a reduction in pain and stiffness, and improved physical function in subjects with knee osteoarthritis pain. TC appears to serve as a viable means for non-pharmaceutical treatment to modify systemic production of pro-inflammatory OxL compounds. Controlled clinical trials are warranted to confirm our findings and expand on the specific changes for both OxL and eCB following TC exercise. Moreover, TC altered levels of OxL derived from n-3 and n-6 PUFA family substrate and decreased pro-inflammatory states. The changes in brain connectivity post-TC and correlations with plasma eCB and OxL levels are noteworthy and suggest an important relationship with neuroinflammation and brain neuroplasticity for these lipid biomarkers under mind–body exercise. Future studies should examine the effects of dietary n-3 PUFA and include dietary records to ascertain if n-3 PUFA substrates alter the types of eCB during TC, and further change pro-inflammatory OxL during TC in a randomized placebo-controlled trial with a larger sample size for an adequate power. Dietary approaches or supplements that lower substrate for arachidonic acid with n-3 PUFA or fish oil may work synergistically to help reduce pain with TC exercise. In this scenario, emphasis must be placed on brain connectivity and plasticity. Hence, we do support the practice of TC to help control pain in women with knee osteoarthritis given the changes in eCB and OxL.

## Data availability statement

The raw data supporting the conclusions of this article will be made available by the authors, without undue reservation.

## Ethics statement

The studies involving humans were approved by Texas Tech University Health Sciences Center Lubbock IRB. The studies were conducted in accordance with the local legislation and institutional requirements. Written informed consent for participation in this study was provided by the participants’ legal guardians/next of kin.

## Author contributions

C-LS, JN, M-CC, CK, VN, and BW: conceptualization and methodology. JN, ME, KB, and CK: data collection and analysis. C-LS, JN, ME, and BW: writing-original draft preparation. C-LS, JN, ME, M-CC, CK, KB, VN, and BW: writing-review and editing. C-LS, M-CC, and VN: supervision, project administration, and funding acquisition. All authors contributed to the article and approved the submitted version.

## Funding

This study was supported by NIH/NINDS R01 NS038261 (VN), Texas Tech University Neuroimaging Institute, Texas Tech University, Lubbock, TX (CLS) and the Center of Excellence for Translational Neuroscience and Therapeutics, Texas Tech University Health Sciences Center, Lubbock, TX (CLS and VN). Additional support was provided by USDA Intramural Projects 2032-51530-025-00D (JN). The USDA is an equal opportunity employer and provider. The funders had no influence on the conceptualization, data collection, analysis, or interpretation of data presented within the manuscript.

## Conflict of interest

The authors declare that the research was conducted in the absence of any commercial or financial relationships that could be construed as a potential conflict of interest.

## Publisher’s note

All claims expressed in this article are solely those of the authors and do not necessarily represent those of their affiliated organizations, or those of the publisher, the editors and the reviewers. Any product that may be evaluated in this article, or claim that may be made by its manufacturer, is not guaranteed or endorsed by the publisher.

## Figures and Tables

**Table:** 

AEA	N-arachidonoylethanolamine
AEA	anandamide
A-EA	arachidonoyl ethanolamide
BPI	brief pain inventory
DHEA	docosahexaenoyl ethanolamide
DGLA	dihomo-gamma-linolenic acid
DGLA-EA	dihomo-gamma-linolenoyl ethanolamide
DHA	docosahexaenoic acid
D-EA	docosatetraenoyl ethanolamide
eCB	endocannabinoids
ECS	endocannabinoid system
EPA	eicosapentaenoic acid
LEA	N-linoleylethanolamine
OEA	oleoylethanolamide
OxL	oxylipins
PEA	palmitoylethanolamide
PUFA	polyunsaturated fatty acids
SEA	N-stearoylethanolamine
VAS	Visual Analog Scale
WOMAC	Western Ontario and McMaster Universities Osteoarthritis Index
αLEA	alpha N-linoleylethanolamine
αL-EA	alpha-linolenoyl ethanolamide
1-AG	1-arachidonoylglycerol
2-AG	2-arachidonoylglycerol
2-LG	2- linoleoylglycerol
1-OG	1-oleoylglycerol
2-OG	2-oleoylglycerol
5-HEPE	5-hydroxy-6E,8Z,11Z,14Z,17Z-eicosapentaenoic acid
8,9-DiHETrE	8,9-dihydroxy-5Z,11Z,14Z-eicosatrienoic acid
8(9)-EpETrE	8(9)-epoxy-5Z,11Z,14Z-eicosatrienoic acid
9(10)-EpODE	9(10)-epoxy-12Z,15Z-octadecadienoic acid
11,12-DiHETrE	11,12-dihydroxy-5Z,8Z,14Z-eicosatrienoic acid
11(12)-EpETrE	11(12)-epoxy-5Z,8Z,14Z-eicosatrienoic acid
12(13)-EpODE	12(13)-epoxy-10E,15Z-octadecadienoic acid
12-HEPE	12-hydroxy-5Z,8Z,10E,14Z,17Z-eicosapentaenoic acid
13-HODE	13-hydroxy-9Z,11E-octadecadienoic acid
13-HpODE	13-hydroperoxy-9Z,11E-octadecadienoic acid
14,15-DiHETE	14,15-dihydroxy-5Z,8Z,11Z,17Z-eicosatetraenoic acid
14(15)-EpETrE	14(15)-epoxy-5Z,8Z,11Z-eicosatrienoic acid
15(16)-EpODE	15(16)-epoxy-9Z,12Z-octadecadienoic acid
17,18-DiHETE	17,18-dihydroxy-5Z,8Z,11Z,14Z-eicosatetraenoic acid
19(20)-EpDPE	19(20)-epoxy-4Z,7Z,10Z,13Z,16Z-docosapentaenoic acid
