# Supplementary material for: Tai Chi exercise reduces circulating levels of inflammatory oxylipins in postmenopausal women with knee osteoarthritis: results from a pilot study
Source: Front Med (Lausanne). 2023 Aug 16;10:1210170. doi: 10.3389/fmed.2023.1210170 (PMC10466388; doi:10.3389/fmed.2023.1210170)
Supplement: Supplementary file 1 [file Table_1.pdf]

Supplementary Materials - Tai Chi exercise reduces circulating levels of inflammatory oxylipins in postmenopausal women with knee osteoarthritis: results from a pilot study

Table S1. Plasma oxylipins at baseline and unaffected by 8wk of Tai Chi (TC) exercise in women with osteoarthritis.

OxL	Substrate fatty acid	Baseline	8 weeks TC	p-value	Standardized difference
6-keto PGF1α	Arachidonic acid	0.05±0.01	0.08±0.03	0.931	0.81
PGF2 α	Arachidonic acid	0.37±0.06	0.50±0.20	0.955	0.80
F2-IsoP	Arachidonic acid	1.17±0.20	1.55±0.30	0.666	1.04
15-deoxy PGJ_2	Arachidonic acid	0.56±0.08	0.68±0.11	0.73	0.93
15-Keto PGE_2	Arachidonic acid	0.86±0.66	2.88±0.80	0.177	1.72
TXB2	Arachidonic acid	5.37±1.44	4.58±0.90	0.963	-0.48
11(12)-EpETE	Eicosapentaenoic acid	1.28±0.26	0.94±0.16	0.489	-1.15
11(12)-EpETrE	Arachidonic acid	0.99±0.15	0.96±0.11	0.796	-0.19
11,12-DiHETrE	Arachidonic acid	0.78±0.13	0.73±0.09	0.931	-0.32
11-HETE	Arachidonic acid	0.66±0.09	0.68±0.10	0.863	0.20
12(13)-Ep-9-KOME	Linoleic acid	1.18±0.33	1.09±0.16	1	-0.27
12(13)-EpODE	α-Linolenic acid	0.29±0.07	0.42±0.08	0.136	1.30
12(13)-EpOME	Linoleic acid	9.36±1.21	12.70±2.41	0.453	1.24
12,13-DiHOME	Linoleic acid	9.61±1.34	11.95±2.99	1	0.71
13-HODE	Linoleic acid	23.66±2.80	27.53±5.55	0.796	0.62
13-HOTE	α-Linolenic acid	1.41±0.20	1.58±0.20	1	0.61
13-KODE	Linoleic acid	8.81±1.04	11.55±1.82	0.489	1.31
14(15)-EpETrE	Arachidonic acid	1.67±0.30	1.67±0.27	0.796	-0.02
14,15-DiHETE	Eicosapentaenoic acid	1.11±0.18	1.09±0.28	0.965	-0.04
14,15-DiHETrE	Arachidonic acid	0.88±0.12	0.85±0.11	0.757	-0.18
14-HDoHE	Docosahexaenoic acid	3.65±0.63	10.79±3.06	0.2	2.15
15(16)-EpODE	α-Linolenic acid	6.21±2.06	4.34±0.38	0.796	-0.89
15,16-DiHODE	α-Linolenic acid	29.17±8.39	17.96±3.52	0.34	-1.23
15-HEPE	Eicosapentaenoic acid	0.20±0.02	0.29±0.12	0.691	0.68
15-HETE	Arachidonic acid	1.06±0.17	1.19±0.20	0.489	0.48
15-KETE	Arachidonic acid	1.67±0.69	1.33±0.27	0.691	-0.39
17(18)-EpETE	Eicosapentaenoic acid	0.39±0.12	0.72±0.32	0.524	1.16
17,18-DiHETE	Eicosapentaenoic acid	4.14±0.71	4.29±0.96	0.93	0.12
17-HDoHE	Docosahexaenoic acid	3.59±0.85	4.42±1.55	0.931	0.47
18-HEPE	Eicosapentaenoic acid	0.01±0.00	0.02±0.01	0.161	1.24
19(20)-EpDoPE	Docosahexaenoic acid	1.29±0.41	1.27±0.19	0.662	-0.04
19,20-DiHDoPE	Docosahexaenoic acid	1.79±0.25	1.92±0.22	0.566	0.40
4-HDoHE	Docosahexaenoic acid	1.23±0.18	0.90±0.24	0.25	-1.09
5,6-DiHETrE	Arachidonic acid	0.55±0.08	0.52±0.08	0.73	-0.31
5-HEPE	Eicosapentaenoic acid	0.84±0.14	0.83±0.21	0.963	-0.06
5-HETE	Arachidonic acid	1.85±0.25	3.01±0.61	0.136	1.76

5-KETE	Arachidonic acid	2.88±0.91	1.71±0.35	0.387	-1.20
6-trans-LTB_4	Arachidonic acid	0.07±0.03	0.29±0.14	0.354	1.95
8(9)-EpETrE	Arachidonic acid	0.68±0.17	0.57±0.17	0.815	-0.43
8,15-DiHETE	Arachidonic acid	0.48±0.11	0.31±0.08	0.19	-1.27
8,9-DiHETrE	Arachidonic acid	0.56±0.12	0.48±0.11	0.436	-0.50
8-HETE	Arachidonic acid	0.81±0.18	1.01±0.17	0.37	0.82
9(10)-EpODE	α-Linolenic acid	1.28±0.24	1.66±0.38	0.666	0.86
9(10)-EpOME	Linoleic acid	3.17±0.55	3.92±1.06	0.743	0.60
9,10-DiHODE	α-Linolenic acid	0.88±0.43	0.55±0.16	0.546	-0.73
9,10-DiHOME	Linoleic acid	11.39±2.79	8.537±2.629	0.387	-0.74
9,10-e-DiHO	Oleic acid	13.32±2.807	12.304±2.91	0.796	-0.25
9,10-EpO	Oleic acid	22.24±5.315	22.663±6.545	0.837	0.05
9,12,13-TriHOME	Linoleic acid	3.81±1.975	1.629±0.537	1	-1.07
9-HEPE	Eicosapentaenoic acid	0.33±0.119	0.356±0.161	0.833	0.13
9-HETE	Arachidonic acid	0.44±0.04	0.52±0.07	0.546	0.94
9-HODE	Linoleic acid	16.30±2.00	16.27±2.88	0.931	-0.01
9-HOTE	α-Linolenic acid	0.66±0.13	0.63±0.09	0.93	-0.18
9-KODE	Linoleic acid	4.98±0.58	4.71±0.94	0.546	-0.24
Lipoxin A4+Epi	Arachidonic acid	4.33±0.96	2.28±0.98	0.13	-1.50
Resolvin E1	Eicosapentaenoic acid	0.02±0.005	0.02±0.003	0.666	-0.84

Units are nM. Values are means ± standard error of the mean of n = 12, baseline and 8-week Tai-Chi intervention in subjects with knee osteoarthritis. *Indicates $p<0.05$. #Indicates $0.05<p<0.1$. Other values are presented as standardized differences calculated from the difference between values of TC treatment and control, divided by the pooled SEM. DiHDoPE = dihydroxy dodecapentaenoic acid DiHODE = dihydroxy octadecadienoic acid; DiHOME = dihydroxy octadecamonoenoic acid; DiHETE = dihydroxy eicosatetraenoic acid; DiHETrE = dihydroxy eicosatrienoic acid; EpDoPE = epoxy dodecapentaenoic acid; EpO = epoxy octadecanoic acid; EpODE = epoxy octadecadienoic acid; EpOME; epoxy octadecamonoenoic acid; EpETE = epoxy eicosatetraenoic acid; EpETrE = epoxy eicosatrienoic acid; EpKOME = epoxy keto octadecamonoenoic aced; HDoHE = hydroxy dodecahexaenoic acid; HEPE = hydroxy eicosapentaenoic acid; HETE = hydroxy eicosatetraenoic acid; HODE hydroxy octadecadienoic acid; HOTE = hydroxy octadecatrienoic acid; KETE = keto eicosatrienoic acid; KODE = keto octadecadienoic acid; LT = leukotriene; PG = prostaglandin; TriHOME = trihydroxy octadecamonoenoic acid.

Table S2. Plasma lipid mediators (Means ± Standard deviations nM), p-values, and VIP scores, in participants before and after 8wk of Tai Chi therapy. Missing values were imputed by Visit if present in >70% of subjects.

Metabolite	Units	Wk 0	Wk 8	p-value	VIP
PGE2	nM	14.8 ± 15	2.13 ± 1.9	0.0204	2.3963
12-HEPE	nM	0.322 ± 0.17	1.95 ± 2.8	0.0003	2.2923
LTB4	nM	0.0379 ± 0.029	0.373 ± 0.33	0.0021	2.2823
LEA	nM	4.81 ± 1.1	3.75 ± 1.9	0.0781	2.2789
13-HpODE screen	Unit less	0.0248 ± 0.016	0.0177 ± 0.02	0.0212	2.0555
8,15-DiHETE	nM	0.479 ± 0.31	0.312 ± 0.24	0.087	1.8553
NA-Gly	nM	1.13 ± 0.79	0.892 ± 1.4	0.2842	1.6954
14-HDoHE	nM	3.63 ± 1.7	10.8 ± 9.2	0.0153	1.5452
AEA	nM	2.68 ± 0.87	2.1 ± 1	0.076	1.5091
12-HETE	nM	6.24 ± 3.2	18.5 ± 16	0.0467	1.4971
Lipoxin A4+Epi	nM	4.04 ± 2.7	2.58 ± 2.7	0.0282	1.4758
NO-Gly	nM	6.17 ± 3.8	4.41 ± 2.5	0.1825	1.4404
Protectin DX	nM	0.163 ± 0.18	0.397 ± 0.25	0.1327	1.433
DHA (Area Ratio)	Unit less	5.36 ± 4.3	5.75 ± 3.7	0.4218	1.3926
9,10-DiHOME	nM	11.4 ± 8.4	8.54 ± 7.9	0.4146	1.3624
6-trans-LTB4	nM	0.0797 ± 0.063	0.248 ± 0.23	0.0383	1.3454
20-HETE	nM	5.47 ± 3.1	7.38 ± 3.5	0.0253	1.3391
9-HpODE screen	Unit less	0.0291 ± 0.021	0.0198 ± 0.013	0.1472	1.315
9,10-e-DiHO	nM	13.3 ± 8.4	12.3 ± 8.7	0.7653	1.3046
8,9-DiHETrE	nM	0.564 ± 0.35	0.484 ± 0.33	0.6589	1.2727
12,13-DiHODE	nM	0.812 ± 1.1	2.37 ± 3.2	0.0496	1.2172
EPA (Area Ratio)	Unit less	5.19 ± 3.9	5.92 ± 3.6	0.3802	1.1974
5-HETE	nM	1.85 ± 0.74	3.01 ± 1.8	0.049	1.1937
OEA	nM	10.6 ± 4.1	8.47 ± 2.1	0.1949	1.1667
2-OG	nM	1260 ± 1100	1440 ± 760	0.3698	1.1329
5,6-DiHETrE	nM	0.552 ± 0.25	0.516 ± 0.24	0.4909	1.1219
4-HDoHE	nM	1.23 ± 0.54	0.899 ± 0.72	0.2887	1.1206
DHEA	nM	1.35 ± 0.65	1.14 ± 0.65	0.1342	1.1051
Acetaminophen	nM	322 ± 930	20 ± 29	0.452	1.0398
18-HEPE	nM	0.0111 ± 0.0092	0.0213 ± 0.023	0.172	1.0287
ALA (Area Ratio)	Unit less	4.99 ± 1.9	6.13 ± 3.3	0.407	0.9908
11(12)-EpETE	nM	1.28 ± 0.76	0.938 ± 0.47	0.3812	0.984
Resolvin E1	nM	0.0239 ± 0.015	0.0191 ± 0.0086	0.4811	0.9417
6-keto PGF1a	nM	0.0526 ± 0.016	0.0994 ± 0.068	0.105	0.9413
19,20-DiHDoPA	nM	1.79 ± 0.74	1.92 ± 0.66	0.5162	0.9323

9,10-DiHODE	nM	0.881 ± 1.3	0.547 ± 0.48	0.2856	0.9209
5-KETE	nM	2.88 ± 2.7	1.71 ± 1	0.4975	0.9145
9-HEPE	nM	0.2 ± 0.24	0.299 ± 0.46	0.3843	0.9024
9-HOTE	nM	0.661 ± 0.37	0.634 ± 0.27	0.6868	0.9006
PEA	nM	6.98 ± 5.5	6.77 ± 3.6	0.9649	0.7906
13-KODE	nM	8.81 ± 3.1	11.6 ± 5.5	0.1777	0.7776
9(10)-EpODE	nM	1.28 ± 0.72	1.66 ± 1.1	0.3242	0.7595
9(10)-EpOME	nM	2.9 ± 1.7	3.92 ± 3.2	0.2505	0.7506
(1+2)-OG	nM	4340 ± 4100	3800 ± 2100	0.5695	0.7218
SEA	nM	3.86 ± 1.2	3.87 ± 1.3	0.9166	0.7128
15,16-DiHODE	nM	29.2 ± 25	18 ± 11	0.4308	0.7082
9-KODE	nM	4.98 ± 1.7	4.71 ± 2.8	0.5162	0.6951
Ibuprofen	nM	54.2 ± 55	86.2 ± 150	0.7255	0.6872
14,15-DiHETrE	nM	0.881 ± 0.34	0.853 ± 0.32	0.8595	0.677
15-deoxy PGJ2	nM	0.559 ± 0.24	0.684 ± 0.32	0.2177	0.6714
12(13)-EpODE	nM	0.288 ± 0.19	0.423 ± 0.25	0.2154	0.6671
8(9)-EpETrE	nM	0.677 ± 0.52	0.607 ± 0.47	0.854	0.6314
POEA Screen	Unit less	0.316 ± 0.13	0.276 ± 0.14	0.3617	0.6236
9,12,13-TriHOME	nM	3.81 ± 5.9	1.63 ± 1.6	0.6983	0.6139
12(13)-Ep-9-KODE	nM	1.32 ± 0.8	1.09 ± 0.47	0.4602	0.6026
12(13)-EpOME	nM	9.36 ± 3.6	12.7 ± 7.2	0.4846	0.5899
11(12)-EpETrE	nM	0.992 ± 0.45	0.957 ± 0.33	0.8915	0.5882
1-OG	nM	3080 ± 3200	2350 ± 1500	0.6803	0.5764
5-HEPE	nM	0.839 ± 0.42	0.8 ± 0.55	0.691	0.5659
9,10-EpO	nM	22.2 ± 16	25.2 ± 21	0.7096	0.5404
13-HOTE	nM	1.41 ± 0.6	1.58 ± 0.6	0.6246	0.5398
9-HODE	nM	16.3 ± 6	16.3 ± 8.6	0.8587	0.5183
15-HETE	nM	1.06 ± 0.52	1.19 ± 0.61	0.6414	0.5093
Naproxen	nM	6800 ± 11000	10600 ± 39000	0.5567	0.5056
17-HDoHE	nM	3.59 ± 2.5	4.42 ± 4.6	0.5729	0.4706
14(15)-EpETrE	nM	1.67 ± 0.89	1.67 ± 0.81	0.9767	0.4697
LA (Area Ratio)	Unit less	5.21 ± 1.5	5.9 ± 2.5	0.4929	0.4659
15(16)-EpODE	nM	6.21 ± 6.2	4.34 ± 1.1	0.9516	0.4548
2-LG	nM	1560 ± 780	1430 ± 770	0.6374	0.4503
9-HETE	nM	0.444 ± 0.13	0.523 ± 0.22	0.5307	0.4445
10-Nitrooleate	nM	5.06 ± 7.3	5 ± 5.3	0.7518	0.4136
15-KETE	nM	1.62 ± 2.1	1.33 ± 0.8	0.6766	0.3961
1-AG	nM	142 ± 130	98.4 ± 42	0.561	0.3951
TXB2	nM	4.66 ± 4.4	4.58 ± 2.7	0.9126	0.3909
17,18-DiHETE	nM	4.14 ± 2.1	4.29 ± 2.9	0.9271	0.3874
8-HETE	nM	0.81 ± 0.54	1.01 ± 0.44	0.1252	0.3666
11,12-DiHETrE	nM	0.779 ± 0.38	0.729 ± 0.27	0.7453	0.3518
(1+2)-AG	nM	245 ± 200	172 ± 74	0.5544	0.3371

14,15-DiHETE	nM	1.1 ± 0.55	1.09 ± 0.82	0.7286	0.3359
17(18)-EpETE	nM	0.365 ± 0.32	0.641 ± 0.56	0.1855	0.3172
1-LG	nM	4110 ± 1900	3430 ± 2300	0.4733	0.3062
2-AG	nM	104 ± 72	73.8 ± 39	0.438	0.2676
F2-IsoP	nM	1.17 ± 0.61	1.55 ± 0.89	0.334	0.2635
15-HEPE	nM	0.202 ± 0.071	0.286 ± 0.37	0.6906	0.263
13-HODE	nM	23.7 ± 8.4	27.5 ± 17	0.89	0.2389
AA (Area Ratio)	Unit less	5.64 ± 2.5	5.47 ± 1.9	0.8996	0.2269
aLEA	nM	0.177 ± 0.046	0.198 ± 0.13	0.8398	0.2255
11-HETE	nM	0.656 ± 0.26	0.683 ± 0.3	0.9973	0.1783
12,13-DiHOME	nM	9.61 ± 4	12 ± 9	0.9578	0.1497
(1+2)-LG	nM	5660 ± 2500	4860 ± 2700	0.5388	0.1187
EPEA Screen	nM	0.063 ± 0.051	0.0576 ± 0.035	0.9892	0.1024
PGF2a	nM	0.365 ± 0.17	0.505 ± 0.39	0.5335	0.0513

Missing values were imputed by Visit if present in >70% of subjects. Mean differences were assessed on data after normal transformation by 2-tailed t-tests. Partial least square discriminate analysis variable importance in projection scores of 3-dimensional analyses (Q2 = 0.64, r2X =0.36; r2y =0.99) are shown. Results are ranked by descending variable importance in projections (VIP) scores.

Abbreviations: 1-AG, 1-arachidonoylglycerol; 1-LG, 1-linoleoyl glycerol; 1-OG, 1-oleoyl glycerol; 2-AG, 2-arachidonoylglycerol; 2-LG, 2-linoleoyl glycerol; 2-OG, 2-oleoyl glycerol; 5-HEPE, 5-hydroxy-6E,8Z,11Z,14Z,17Z-eicosapentaenoic acid; 8,9-DiHETrE, 8,9-dihydroxy-5Z,11Z,14Z-eicosatrienoic acid; 8(9)-EpETrE, 8(9)-epoxy-5Z,11Z,14Z-eicosatrienoic acid;
9(10)-EpODE , 9(10)-epoxy-12Z,15Z-octadecadienoic acid; 11,12-DiHETrE, 11,12-dihydroxy-5Z,8Z,14Z-eicosatrienoic acid; 11(12)-EpETrE, 11(12)-epoxy-5Z,8Z,14Z-eicosatrienoic acid;
12(13)-EpODE, 12(13)-epoxy-10E,15Z-octadecadienoic acid; 12-HEPE, 12-hydroxy-5Z,8Z,10E,14Z,17Z-eicosapentaenoic acid; 13-HODE, 13-hydroxy-9Z,11E-octadecadienoic acid; 13-HpODE, 13-hydroperoxy-9Z,11E-octadecadienoic acid; 14,15-DiHETE, 14,15-dihydroxy-5Z,8Z,11Z,17Z-eicosatetraenoic acid; 14(15)-EpETrE, 14(15)-epoxy-5Z,8Z,11Z-eicosatrienoic acid; 15(16)-EpODE, 15(16)-epoxy-9Z,12Z-octadecadienoic acid; 17,18-DiHETE, 17,18-dihydroxy-5Z,8Z,11Z,14Z-eicosatetraenoic acid; 19(20)-EpDPE, 19(20)-epoxy-4Z,7Z,10Z,13Z,16Z-docosapentaenoic acid; αLEA, alpha-linolenoyl ethanolamide; AEA, arachidonoyl ethanolamide; AEA, anandamide; D-EA, docosatetraenoyl ethanolamide; DAGL, diacylglycerol lipase; DGLA, dihomo-gamma-linolenic acid; DGLA-EA, dihomo-gamma-linolenoyl ethanolamide; DHEA, docosahexaenoyl ethanolamide; DHA, docosahexaenoic acid; EPA, eicosapentaenoic acid; LEA, linoleoyl ethanolamide; OEA, oleoyl ethanolamide; PEA, palmitoyl ethanolamide; SEA, stearoyl ethanolamide.
